# Metastatic Prostate Cancer to the Optic Canal: A Large-Cohort Analysis

**DOI:** 10.7759/cureus.46598

**Published:** 2023-10-06

**Authors:** Neil Kelkar, Dawood Findakly, Jue Wang

**Affiliations:** 1 Oncology, University of Arizona College of Medicine - Phoenix, Phoneix, USA; 2 HemeOnc, Louisiana State University Health Sciences Center Shreveport, Shreveport, USA; 3 Division of Hematology and Oncology, Department of Internal Medicine, University of Texas Southwestern Medical Center, Dallas, USA

**Keywords:** anti-androgen therapy, metastatic prostate cancer, prostate cancer, orbital metastasis, optic canal metastasis

## Abstract

Prostate cancer (PCa) can present with metastases in rare cases, including those to the optic canal. Currently, no guidelines exist for managing PCa metastases in this patient population. This article aims to examine optic canal metastases through a large-cohort analysis. It involves a systematic review of the literature through a pooled analysis of published cases of PCa with optic canal or orbital metastasis, including one case previously reported from our institution. A subgroup analysis was employed to compare cases with optic metastases as their initial PCa presentation, hormone-naive (HN), versus those with optic metastases after PCa diagnosis, hormone-refractory (HR).

A total of 45 patients with ocular metastasis from PCa were included in this study. The mean age at diagnosis overall was 66.8 years (range: 45-85 years). Moreover, 16 cases (50%) had deceased, with a median time-to-death of 22 (range: 2-84) months. Regarding subgroup analysis, the mean age at diagnosis was 69.5 years (53-85 years) in the HN group and 64.5 years (45-83 years) in the HR group. With regard to reported survival, 10 (62.5%) cases belonged to the HN subset with a median follow-up duration of 12 (range: 1.5-36) months. In terms of reported mortality, 10 (62.5%) were from the HR subset with a median time-to-death of 32.5 (range 0.5-84) months. Our study constitutes the largest and most comprehensive examination of patients with optic canal metastases due to PCa so far. While optic canal metastases are a rare manifestation of PCa, they are linked with a poor prognosis. We also observed significant differences between HR and HN cohorts, which may indicate a difference in clinical presentations.

## Introduction and background

Prostate cancer (PCa) is the second most common cancer, affecting millions of men globally, and the second leading cause of cancer-related deaths among males in the United States. Patients with a localized or regional PCa have a favorable prognosis with a five-year overall survival rate of more than 99% compared to only 30% in patients presenting with metastatic disease [1]. It has been that there has been an increase in metastatic PCa incidence since the United States Preventive Services Task Force (USPSTF) recommended against screening for PCa for men aged 75 years and older in 2008 and all men in 2012. The rate of distant-stage diagnoses has more than doubled, from about 4% in 2007 to more than 8% in 2018 [2].

PCa commonly metastasizes to the spinal canal, axial bones, and brain. However, although less common, PCa is also reported to present with ocular metastases. It has been suggested that ocular malignancies are not as rare as they appear, and in approximately 20% of patients, orbital metastases can be the initial manifestation of systemic malignancy [3]. Ocular malignancies can be either from primary sources or through secondary malignancies (such as leukemia or distant metastases). Although the two most common sites of ocular metastasis are the choroid and the orbit, rare cases of optic metastases have been documented [4]. One such documented ocular cancer is that of the optic canal by PCa.

In this study, we present the largest case series so far of patients with rare optic canal metastases due to PCa. Our study identified 45 cases of metastatic PCa with optic canal involvement reported in the literature [4-30]. We engage in an analysis of their clinical characteristics, notable histology, and treatment modalities. The study also involves a comparison between 20 cases (44%) of hormone-naïve (HN) PCa and 25 cases (56%) of hormone-refractory (HR) PCa. There are no established guidelines for managing patients with this rare metastasis. In light of this, our study aims to document significant clinical correlates pertaining to this condition based on a large data subset.

## Review

Methodology

Literature Search

**Figure 1:**
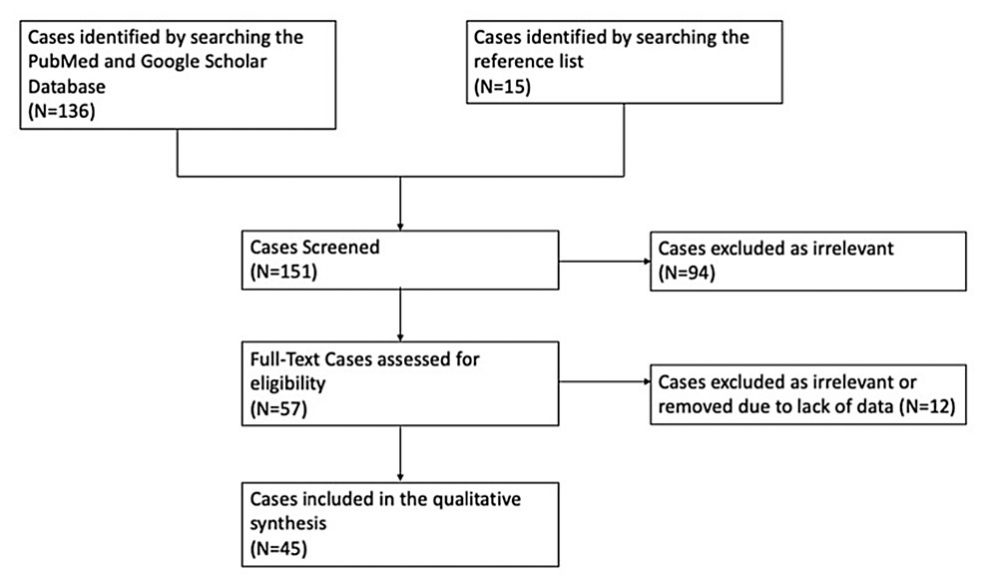
PRISMA flowchart depicting the selection of cases PRISMA: Preferred Reporting Items for Systematic Reviews and Meta-Analyses

A systematic review of the literature was performed by searching the PubMed and Google Scholar databases by using the following terms: “optic canal metastasis,” “optic canal prostate metastasis,” and “prostate cancer.” The identified case reports and associated papers were reviewed for eligibility of relevant material. Studies not involving prostate cancer or optic canal tumors were excluded. Ultimately, 45 cases were selected for the final analysis, as illustrated in the Preferred Reporting Items for Systematic Reviews and Meta-Analyses (PRISMA) flowchart in Figure 1.

Data Collection and Analysis

Data related to patients (age at diagnosis, presentation, associated medical history), tumors (histology, grade), laboratory results (PSA), treatment (PCa and optic canal treatment), radiographic results, and outcomes (response, adverse effects, mortality) were recorded when available. Descriptive statistics were used to assess the pooled sample. Patients were then subsequently divided into two groups (HN and HR) based on their clinical history and compared in terms of significant differences, followed by further assessment of individual pooled samples.

Results

Patient Characteristics

A total of 45 cases of metastatic PCa with optic canal involvement were identified from 26 different case reports and five case series. One of the cases was previously reported from our institution. The mean age at diagnosis (±SD, range) was 66.8 (±8.4, 45-85) years. Among cases where prostate-specific antigen (PSA) was documented, 14 had elevated PSA. The median recorded PSA was 151.5 ng/mL for the overall cohort. Of note, 32 cases reported outcomes data, with an overall median follow-up period of 13.5 (range: 0.5-84) months. At the last follow-up, 16 (50%) patients were alive, and the median follow-up period among them was 12 (range: 0.5-60) months. Moreover, 16 cases (50%) had deceased, with a median time-to-death of 22 (range: 2-84) months.

**Figure 2:**
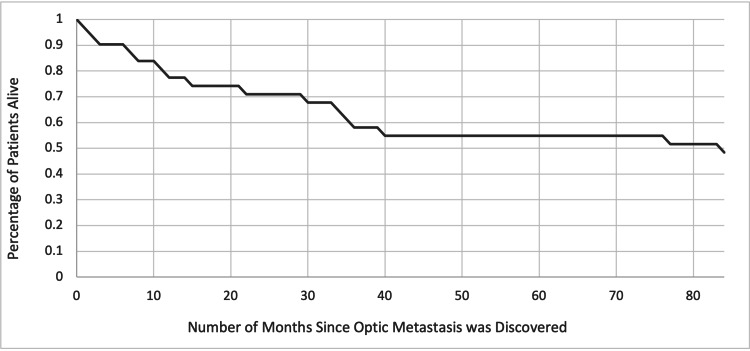
Survival curve of patients with optic canal metastases due to prostate cancer

Among patients with metastatic PCa, 20 (44%) presented with optic metastases at the time of their initial presentation (HN) of PCa, and 25 patients (56%) presented with optic metastases after their established diagnosis (HR) of PCa. The mean age in the HN group was 69.5 (range: 53-85) years, while that in the HR group was 64.5 (range: 45-84) years. Cases that reported PSA values were equally split between HN and HR subsets, and five cases were noted to have normal PSA: all were from the HR subset (HN = 140 ng/mL, HR = 115 ng/mL). Out of 16 patients who survived, 10 (62.5%) were from the HN subset, with a median follow-up duration of 12 (range: 1.5-36) months. Out of 16 cases who deceased, 10 (62.5%) were from the HR subset, with a median time-to-death of 32.5 (range: 0.5-84) months. A breakdown of patient characteristics within the two subgroups is presented in Table 1. A survival curve is depicted in Figure 2.

**Table 1:** Breakdown of patient characteristics *Statistically significant

Variables	Initial-presentation subset	After-diagnosis subset	Overall	P-value
Number of cases	20	25	45	
Average age, years	69.5	64.5	66.8	0.0767*
Median PSA, ng/mL	140	115	151.5	0.9197
Survival, n (%)	10 (62.5%)	6 (37.5%)	16 (50%)	0.0351*
Bony metastases, n (%)	13 (65%)	10 (40%)	23 (51%)	0.0477*

Metastatic sites varied between cases, with all cases (100%) from the HN group reporting additional metastases while 20 cases (80%) from the HR group did the same. Twenty-three cases (49%) presented with widespread lesions involving the axial skeleton: 13 HN cases (65%) and 10 HR cases (40%). Six cases (13%) reported involvement with bilateral ocular structures, with three in the HN subset and three in the HR subset. Thirteen cases (29%) involved additional metastases to the optic nerve, optic disc, choroid, or orbit, with five HN cases (25%) versus eight HR cases (32%). One case (2%) involved bilateral solitary lesions to the optic nerves, and it was part of the HN subset.

The common complaints that patients presented with were as follows: proptosis (18 cases, 40%), diplopia (10 cases, 22%), decreased visual acuity (21 cases, 47%), and progression to vision loss (15 cases, 33%). Eighteen cases (40%) also reported additional systemic symptoms, including pain (14 cases, 31%) and specific urinary complaints (three cases, 7%). Only one case was reported not to have any associated symptoms from PCa. A breakdown of these symptoms is shown in Table 2.

**Table 2:** Breakdown of reported ocular symptoms and findings *Statistically significant

Symptom reported	Initial-presentation subset, %	After-diagnosis subset, %	P-value
Decreased visual acuity	45	48	0.421
Proptosis	28	55	0.0365*
Vision loss	20	44	0.0484*
Diplopia	35	12	0.0360*
Pain (unspecified location)	40	24	0.129
Periorbital swelling	20	4	0.0485*
Retinal findings	20	4	0.0485*
Additional ocular metastases	5	8	0.3033

Histology

**Figure 3:**
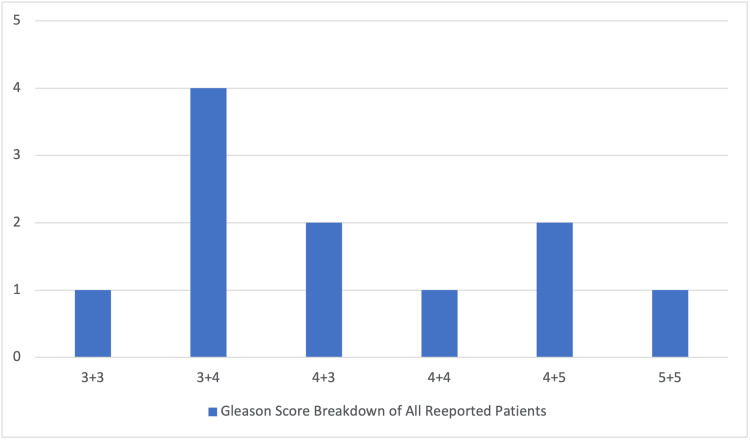
Breakdown of reported Gleason scores

Of the cases with reported histology, 40 (97.5%) involved prostate adenocarcinoma, and one (2.5%) reported small-cell prostate carcinoma, which was part of the HR subset. Of the total cases, 26 included a histological description of the differentiation of cancerous cells (13 HN vs. 13 HR). Of these, six cases were documented as poorly differentiated (three HN vs. three HR), and five cases were documented as moderately/well differentiated (four HN vs. one HR). There were 16 cases with documented PCa staging (zero HN vs. five HR) or Gleason scores (seven HN vs. four HR). Among the cases with reported Gleason scores, one case was 3+3 (one HN vs. zero HR), four cases were 3+4 (two HN vs. two HR), two cases were 4+3 (two HN vs. zero HR), one case was 4+4 (one HN vs. zero HR), two cases were 4+5 (one HN vs. one HR), and one case was 5+5 (zero HN vs. one HR). A breakdown of total Gleason scores is illustrated in Figure 3.

Treatment

Treatment for the PCa involved 17 instances of androgen deprivation (five HN vs. 12 HR), six cases of steroid use (one HN vs. five HR), 16 cases of external beam radiation (four HN vs. 12 HR), and four cases of debulking procedures (two HN vs. two HR). Older data reported 19 cases of bilateral orchiectomies (eight HN vs. 11 HR) and five cases of diethylstilbestrol (DES) use (zero HN vs. five HR).

Treatment for optic canal metastases included 10 instances of steroid use (nine HN vs. one HR), 13 cases of radiation (11 HN vs. two HR), five cases of anti-androgen therapy (four HN vs. one HR), and six cases of ocular surgical intervention (zero HN vs. six HR). It should be noted that there were nine cases without any therapy (two HN vs. seven HR).

Discussion

PCa is the leading cause of orbital metastases in male patients, followed by lung, melanoma, kidney, and gastrointestinal cancers [20]. Unfortunately, PCa metastases to the eye have a higher mortality rate compared to other cancers [20]. The ocular oncology service at Wills Eye Hospital in Philadelphia has previously reported that in a case series of 100 patients with orbital metastases, only 19% had no history of cancer at the time of presentation [20]. In our sample, we found the rate of initial diagnosis of PCa with optic canal metastases to be at 44%.

Our study constitutes the largest cohort analysis examining optic canal metastases specifically. The study outlines several notable findings. Firstly, the HN and HR subsets had notable differences in terms of optic nerve compression, with the HR subset having a significantly higher incidence rate (Table 1). This is perhaps due to the insidious onset of vision loss in this subset, given the failure of the initial treatment of PCa [5]. Secondly, death from PCa was higher in the HR group, which is also perhaps linked to primary treatment failure. However, the possibility of several patients opting for palliative therapy, patients declining treatment, and loss to follow-up should also be considered. Despite an increase in the rate of early prostate cancer screening, patients can still present with distant metastasis at the time of their initial presentation.

Our findings also indicated that histological results differed between the groups. Most PCa (95%) cases were adenocarcinoma, with only 5% being of small-cell origin. The study results were similar in terms of how common the degree of differentiation was in the overall cohort. Patients who presented with HR optic canal metastases were more likely to have poorly differentiated PCa compared to their HN counterparts. This trend was mirrored in the analysis of Gleason scores, where low grades were more likely in the HN subset while high grades were more likely in the HR subset.

It should be noted that ocular metastases from PCa have a greater mean age of onset than other ocular metastases. One study reported a mean age of 70.1 years for PCa versus 53.6 years for other ocular metastases [4]. Our results indicate a slightly lower rate at 66.8 years for PCa, with the HN subset having a mean age of 69.5 years. This suggests that older male patients who are noted to have a mass in the optic canal should be evaluated to rule out metastasis from PCa, given its high reported incidence.

Typical clinical findings included limitations in ocular motility, proptosis, and palpable ocular masses; however, the specific symptoms differed as to whether the metastases were anterior or posterior on the orbit [5,20]. Our results indicate that the HN subset was more likely to present with diplopia secondary to a limitation in ocular motility, and the HR subset was more likely to present with vision loss. The mechanism of decreased visual acuity and vision loss in these patients is likely due to optic nerve compression [15]. Orbital MRIs examined by Kattah et al. showed focal enlargement and contrast enhancement in the optic nerve sheath of affected patients, in addition to ischemic changes and optic nerve atrophy [5].

There is currently no optimal treatment modality for managing patients with this presentation, and prompt treatment from oncologists is essential for patients. The treatment of patients in our cohort involved using androgen deprivation, radiation, and steroid use. Additional treatment modalities involved the use of orchiectomies for PCa. There were differences between the groups in terms of treatment as well. Patients with a history of PCa were more likely to be provided external beam radiation, androgen deprivation, and other chemotherapeutic medications (e.g., DES) to manage their condition. However, the HN PCa population was more likely to undergo surgical interventions to manage their condition. The treatment for specific optic metastases also involved using steroids, radiation, and surgical intervention for ocular complications. The cohort received treatment with androgen deprivation and steroids overall, which could help prevent optic nerve compression. Treatment with radiation was more likely to be reported in the HN cohort, probably due to having a more treatable manifestation. It should be noted that endocrine treatment and external beam radiotherapy are considered the gold standard methods for treating these ocular metastases [20]. Additional treatment also included enucleation of the involved eye, which was significantly more likely in the HR cohort.

Palliative treatments were likely necessary given the late stage of this disease. Patients who presented with optic canal metastases at their initial presentation were more likely to receive ocular surgical interventions, likely to help reach a diagnosis. It should be noted that diagnosing PCa in these scenarios can be challenging, as PSA can be expected, as was the case for four patients in this study who were undergoing treatment for their PCa [6,7,11,24]. The median time-to-death from ocular metastasis from PCa was 22 (range: 2-84) months. It should be noted that despite utilizing all available treatments, the mortality rate is still high. This highlights an urgent need to develop newer treatment modalities for PCa.

This study has a few limitations, primarily due to the rarity of this specific metastasis. These include the possibility of misleading conclusions that may change with an increasing number of samples; readers should exercise caution when drawing specific conclusions. However, this study provides the most up-to-date information on this specific metastasis despite the small sample size.

## Conclusions

To our knowledge, this is the largest review of the subset of PCa patients with optic canal metastases so far. Optic canal metastases are a particularly rare manifestation of PCa. However, studies examining metastatic ocular metastases have indicated that approximately half of all ocular metastases are from PCa. Our findings showed that optic canal metastases are associated with an unfavorable prognosis in general, like other ocular PCa metastases. Furthermore, we noted certain significant differences between the HR and HN cohorts, including a higher Gleason score for the HR cohort with worse survival outcomes when compared to the HN cohort. The treatment for this entity requires prompt management, preferably involving a multidisciplinary team, to achieve favorable outcomes.
